# The relationship between spiritual health and COVID-19 anxiety among nurses: a national online cross-sectional study

**DOI:** 10.1038/s41598-024-67523-7

**Published:** 2024-07-16

**Authors:** Arezoo Davarinia Motlagh Quchan, Fatemeh Mohammadzadeh, Zohreh Mohamadzadeh Tabrizi, Narjes Bahri

**Affiliations:** 1 Department of Health in Emergencies and Disasters, School of Public Health, Tehran University of Medical Sciences, Tehran, Iran; 2 Department of Epidemiology & Biostatistics, School of Health, Social Development & Health Promotion Research Center, Gonabad University of Medical Sciences, Gonabad, Iran; 3 Department of Nursing, School of Nursing and Midwifery, Mashhad University of Medical Sciences, Mashhad, Iran; 4 Department of Midwifery, Faculty of Medicine, Social Determinants of Health Research Center, Gonabad University of Medical Sciences, Gonabad, Iran

**Keywords:** COVID-19, Anxiety, Spirituality, Nurses, Iran, Psychology, Health care, Medical research

## Abstract

The Coronavirus disease 2019 (COVID-19) outbreak has created many concerns in most countries. Nurses are among healthcare workers who are largely engaged in providing care to COVID-19 patients, which makes nurses prone to disease-related worries and stresses. Thus, it is essential to identify the factors which may alleviate their stress and anxiety. This study aimed to determine the relationship between COVID-19 anxiety and spiritual health among Iranian nurses. This cross-sectional online survey was conducted between March 2020 and January 2021 on 919 Iranian nurses who worked in healthcare centers in Iran during the COVID-19 outbreak. The participants were recruited by convenience sampling Method. The data were collected using a demographic questionnaire, Ellison’s standard Spiritual Well-Being (SWB) Scale, and the standard scale of Corona Disease Anxiety. Data were analyzed using SPSS software and p-value less than 0.05 was considered significant. Of the participants, 47.0% (95% confidence interval (CI) 43.7–50.2%) had moderate to severe anxiety. The mean score of spiritual health was 73.3 ± 12.5. The multiple linear model indicated a significant negative correlation with a medium effect size between SWB and COVID-19 anxiety levels in a way that for 10 units increase in the SWB was associated with 2.72 units decrease in anxiety score (Adjusted partial r = − 0.320, *p* < 0.001). The findings revealed a reverse significant relationship between spiritual health and COVID-19 anxiety. On the other hand, nurses with better spiritual health experienced a lower level of COVID-19 anxiety. Therefore, improving spiritual health could help decrease nurses’ anxiety during COVID-19.

## Introduction

Coronaviruses are a large family of viruses that cause respiratory infections, and induce severe diseases such as Middle East Respiratory Syndrome (MERS) and severe acute respiratory syndrome (SARS). The Coronavirus Disease 2019 (COVID-19) outbreak began on December 19th, 2019 in Wuhan, China, and rapidly spread across the world^1,2^. The rise in the number of affected individuals and the global spread of this virus, the World Health Organization (WHO) issued a statement on January 11th, 2020, and declared the COVID-19 outbreak the sixth public health emergency of international concern that threatened not only China, but all the world^3^.

Studies have shown that anxiety symptoms frequently occur in epidemics and pandemics. The symptoms of this type of anxiety may include fear of health-related, social, and economic consequences^4,5^. Studies have shown that COVID-19-related anxiety is prevalent and is mostly due to the unknown nature of the disease and the ambiguities surrounding it^4,6^. Spiritual health is among the important factors that can influence the level of stress, anxiety, and psychological well-being^7^. In times of crisis, spirituality is a powerful source of reducing anxiety^8^.

Spiritual health, along with biological, mental, and social health comprise the four dimensions of human health^9^. Without spiritual health, complete human health may not be achieved^10^. Spiritual health is defined as individual’s spiritual experience in religious and existential health. While existential health focuses on people’s psychosocial concerns and discusses how people adapt to themselves, society, or the environment, spiritual health affects individual’s perception of health within an spiritual context^11^. There is existing evidence about the relation between spiritual health and psychological well-being. For instance, Alemdar et al. reported that spiritual care significantly affected the stress levels of mothers in the Neonatal Intensive Care Unit^12^. Another study showed that spiritual intervention reduced anxiety level in the caregivers of patients with heart failure^13^. Kyivan et al. underlined positive effects for spiritual health on the reduction of pain intensity in burn patients^14^. Implementing supportive spiritual intervention has also been found effective in enhancing mental health and reducing the pain among cancer patients^15^.

Nurses are among the important members of the healthcare team and play a major role in providing care for COVID-19 patients^16^. Direct contact with COVID-19 patients due to occupation requirements, makes nurses prone to COVID-19 anxiety and thus exacerbate their stress and anxiety^16,17^. COVID-19 anxiety in nurses can negatively impact their care-related performance^18^, weaken their immune system, and predispose them to diseases^19,20^. Furthermore, stress and anxiety among nurses can reduce life satisfaction, responsibility, and quality of patient care^21,22^. Therefore, focusing on the spiritual health and maintaining their health, first as human beings and then as individuals in charge of community health, is of great importance^23^.

Due to the COVID-19 pandemic and the resulting anxiety, and because of the important role of nurses in providing care for patients, it is essential to pay attention to strategies to reduce anxiety among nurses. The present study aimed to determine the relationship between COVID-19 anxiety and spiritual health in Iranian nurses.

## Method

### Study design, participants,

This cross-sectional study was conducted on Iranian nurses during March 2020 and January 2021.

The inclusion criteria were as follows: (1) working in hospitals and health service centers in Iran at the time of the COVID-19 outbreak, (2) Iranian nationality, (3) having access to virtual platforms such as Telegram, WhatsApp, and Instagram, and (4) willingness to participation the study.

### Sample size

The following sample size formula was used to determine the sample size for this study considering confidence level of 95%, a test power of 0.9, and a small effect size of E = 0.1 based on the Cohen’s guidelines^24^:

$${\left( {\frac{{{Z_{1 - \alpha /2}} + {Z_{1 - \beta }}}}{E}} \right)^2}$$

The minimum sample size was calculated as 850 nurses. According to Cohen’s guidelines, a confidence level of 95%, a test power of 0.9, and a small effect size of E = 0.1 were inserted in the formula^24^. Considering the possible attrition rate of 10%, the final sample size was increased to 935 nurses.

### Sampling method

The participants were selected based on convenience sampling method. After developing online questionnaires, a link to the online questionnaire was sent to registered Iranian nurses with available cell phone numbers via text messages and related nurse’s groups in social media (Telegram, WhatsApp, and Instagram). Nurses were asked to fill up the online questionnaire and forward the questionnaire to other Iranian nurses they knew. The participants were provided with the contact details of the first author in case of any questions or ambiguity in the questionnaires.

### Study instruments

#### Demographic checklist

This checklist was used to collect individual characteristics such as age, gender, educational level, income level, work experience, smoking, working in Special COVID-19 Department, exposure to COVID-19 patients, history of chronic diseases, mental disorders, and physical exercise. Participants were asked to fill in the required information in the demographic checklist.

#### Ellison's spiritual well-being scale (SWBS)

This scale was introduced by Paloutzian and Ellison in 1982. This scale includes 20 items and includes two dimensions, namely existential well-being (EWB) and religious well-being (RWB). SWBS items are scored on a 6-point Likert scale from “strongly agree” to “strongly disagree”. The scale Positive statements are scored from 1 indicating “strongly disagree” to 5 indicating “strongly agree”, while reverse scoring is applied to negative statements. The EWB and RWB subscales scores may range between 10 and 60 for, and the SWBS total score may range from 20 to 120 for^25^.

The validity and reliability of the Persian version of SWBS were confirmed in the study by Biglari Abhari et al., among a population aged above 18 years in Tehran. The construct structure was discovered and confirmed using exploratory factor analysis and confirmatory factor analysis, respectively. The scale reliability was confirmed using Cronbach's alpha (Cronbach’s alpha of 0.84 for the whole scale, and 0.84, 08.1 for EWB and RWB, respectively)^25^. The reliability of this scale was also evaluated in the present study was examined using Cronbach’s alpha, which was 0.87, 0.89, and 0.92 for EWB, RWB, and total SWBS, respectively.

#### The corona disease anxiety scale (CDAS)

CDAS was developed and validated in Iran by Alipour et al.^16^. CDAS is used to assess anxiety level during the COVID-19 outbreak. The final version of CDAS has 18 items and two dimensions, including psychological symptoms (items 1 to 9) and physical symptoms (items 10 to 18). The items are scored on a 4-point Likert scale including “never” (0), “sometimes” (1), “most of the time” (2), and “all the time” (3). Therefore, the scores may range from 9 to 54. Higher scores indicate a higher level of anxiety.

The reliability of this instrument was evaluated using Cronbach’s alpha, which was 0.879 for the first factor, 0.861 for the second factor, and 0.919 for the total scale. Moreover, Guttman's lambda-2 was 0.882 for the first factor, 0.864 for the second factor, and 0.922 for the total scale. The criterion validity of the scale was evaluated against the GHQ-28. The results showed that the correlation between the scores of CDAS and the total score of the GHQ-28 and the scores of the components of anxiety, physical symptoms, social dysfunction, and depression were respectively 0.483, 0.507, 0.418, 0.269, and 0.333, and all these coefficients were significant at the 0.01 level^20^. The reliability of this scale in the present study was examined by Cronbach’s alpha, which was 0.92, 0.89, and 0.94 for the psychological symptoms, physical symptoms, and the entire scale, respectively.

### Ethical considerations

All the ethical considerations, such as the confidentiality of the data, obtaining informed consent for completing the questionnaire, and the right to withdraw from the study at any time were taken into account. The protocol for this study was approved by the Ethics Committee of the Gonabad University of Medical Sciences (code: IR.GMU.REC.1399.008).

### Statistical analysis

Data were analyzed using SPSS software version 16.0. The normality distribution of quantitative variables was assessed using the Kolmogorov–Smirnov (K–S) test and skewness and kurtosis values. The absolute values greater than 2 for skewness and 7 for kurtosis were considered a severe violation of the normality assumption^26^. Mean (standard deviation [SD]) was used to describe normally distributed quantitative variables, and median (1st, and 3rd quartiles) was used for non-normally distributed quantitative variables.

To provide descriptive statistics on qualitative variables number (percentage) was used. The bivariate correlation between SWB and CDAS and its dimensions was examined using the Pearson correlation coefficient test. A standard multiple linear regression model was used to investigate the relationship between COVID-19 anxiety and SWB. Confounders were identified based on a p-value less than 0.15 in univariate linear regression models. The multivariate model was adjusted for confounders, including age, educational level, income, physical exercise, exposure to COVID-19 patients, history of mental illness, underlying disease (which were selected). An adjusted partial correlation coefficient (adjusted partial r) was used to estimate the effect size. The r values between 0.1 to 0.3, 0.3 to 0.5, and 0.5 to 1.0 were considered as small, medium, and large effect sizes, respectively^27^.

Normality of residuals, the scatter plot of the standardized residuals versus the standardized predicted values, the time series plot of the residuals, and variance inflammation factor (VIF) were used to assess the assumptions of the linear regression model, including normality, homogeneity of variance, independence of errors, and multicollinearity, respectively. The significance level was considered as p value less than 0.05.

## Results

### Demographic characteristics

The data of 919 Iranian nurses were analyzed. The mean age of the participants was 31.3 (SD = 7.0) (range: 20–56) years. Their mean work experience was 7.6 (SD = 6.4) years. The other demographic characteristics are given in Table 1.

**Table 1:** Characteristics of the participants.

Variable	
Age (years), mean (SD)	31.3 (7.0)
Work experience (years), mean (SD)	7.6 (6.4)
Gender, n (%)	
Male	225 (24.5)
Female	694 (75.5)
Educational level, n (%)	
Associate's degree/Bachelor's degree	835 (90.9)
Master's degree/PhD	84 (9.1)
Marital status, n (%)	
Married	585 (63.7)
Single/divorced/widowed	334 (36.3)
Income, n (%)	
Low	332 (36.1)
Moderate and higher	587 (63.9)
Field of expertise, n (%)	
Nursing	701 (76.2)
Anesthesiology	121 (13.2)
Surgical technologist	97 (10.6)
Exposure to COVID-19 patients, n (%)	
Yes	787 (85.6)
No	132 (14.4)
History of mental illness, n (%)	
Yes	72 (7.8)
No	847 (92.2)
Underlying disease, n (%)	
Yes	295 (32.1)
No	624 (67.9)
Smoking cigarettes/hookah, n (%)	
Yes	201 (21.9)
No	718 (78.1)
Physical exercise, (min/week), median $($1st quartile, 3rd quartile)	72.9 (2.0, 11.0)

SD, standard deviation.

### Spiritual well-being

The mean score of SWBF, and EWB and RWB dimensions were 73.3 (SD = 12.5), 33.3 (SD = 7.5), and 40.0 (SD = 6.5), respectively.

### Anxiety severity level concerning COVID-19

The mean total score of CDAS and physical symptoms and psychological symptoms domains were 18.0 (SD = 10.6), 12.9 (SD = 5.7), and 5.6 (SD = 5.1), respectively. Mild, moderate, and severe anxiety was identified in 53.0% (95% CI 49.7–56.2%), 33.3% (95% CI 30.2–36.4%), and 13.7% (95% CI 11.5–16.1%) of the nurses, respectively. Mild, moderate, and severe physical symptoms were identified in 34.8% (95% CI 31.7–37.9%), 47.1% (95% CI 43.8–50.4%), and 18.1% (95% CI 15.6–20.7%) of the nurses, respectively. Mild, moderate, and severe psychological symptoms were identified in 8.2% (95% CI 6.4–10.1%), 78.2% (95% CI 75.4–80.8%), and 13.6% (95% CI 11.4–15.9%) of the nurses, respectively.

### Relationship between spiritual health, individual characteristics, and anxiety severity levels during the COVID-19 outbreak

According to the findings of the Pearson correlation coefficient, there was a negative correlation between the total CDAS score and its physical, and psychological symptoms dimensions, and the total SWB and its EWB and RWB dimensions (Table 2).

**Table 2:** The Pearson correlation coefficients between COVID-9 anxiety and spiritual well-being, and their dimensions.

Variable	Psychological symptoms		Physical symptoms		COVID-19 anxiety (CDAS total score)	
	r	*p*	r	*p*	r	*p*
Existential well-being (EWB)	− 0.367	< 0.001	− 0.349	< 0.001	− 0.383	< 0.001
Religious wellbeing (RWB)	− 0.212	< 0.001	− 0.199	< 0.001	− 0.220	< 0.001
Spiritual well-being (SWBS total score)	− 0.329	< 0.001	− 0.311	< 0.001	− 0.342	< 0.001

A negative and significant relationship was found between SWB and CDAS after adjusting for potential confounding variables, including age, educational level, income, physical exercise, exposure to COVID-19 patients, history of mental illness, and underlying disease, which were selected based on a *p*-value less than 0.15 in univariate linear regression (Table 3). The adjusted partial r for the SWB and COVID-19 anxiety relationship was − 0.320, indicating a medium effect size (*p* < 0.001). For each 10-unit increase in the mean of SWB, COVID-19 anxiety reduced by 2.78 units (B = 0.278, *p* < 0.001).

**Table 3:** Associated risk factors for anxiety severity level during the COVID-19 outbreak based on the results of linear regression model.

Predictors	Simple linear regression			Multiple linear regression		
	B	SE	P	B	SE	P
Age	− 0.072	0.05o	0.150*	− 0.063	0.051	0.218
Gender						
Male	− 3.060	0.811	< 0.001*	− 3.362	0.765	< 0.001**
Female	–	–	–	–	–	–
Educational level						
Associate's degree/Bachelor's degree	2.245	1.217	0.065*	1.943	1.153	0.092
Master's degree/PhD	–	–	–	–	–	–
Marital status						
Married	− 0.144	0.730	0.006*	− 1.181	0.738	0.110
Single/widowed/divorced	–	–	–	–	–	–
Income						
Low	2.563	0.726	< 0.001*	1.260	0.698	0.071
Moderate and higher	–	–	–	–	–	–
Underlying disease						
Yes	2.465	0.748	0.001*	1.769	0.727	0.015**
No	–	–	–	–	–	–
History of mental illness						
Yes	3.883	1.301	0.003*	1.296	1.233	0.293
No	–	–	–	–	–	–
Direct exposure to COVID-19 patients						
Yes	2.394	0.998	0.017*	− 1.992	0.940	0.034**
No	–	–	–	–	–	–
Smoking cigarettes/hookah						
Yes	0.695	0.849	0.414	–	–	–
No	–	–	–	–	–	–
Physical exercise	− 0.006	0.002	0.003*	− 0.003	0.002	0.053
Spiritual health	− 0.290	0.026	< 0.001*	− 0.278	0.027	< 0.001**

SE, standard error; * < 0.15; ** < 0.05.

Furthermore, according to the findings of multiple linear regression, female gender (*p* < 0.001), underlying disease (*p* = 0.015), and direct exposure to COVID-19 patients (*p* = 0.034) were the risk factors for experiencing more severe anxiety during COVID-19 outbreak.

The adjusted R^2^ for the model was 0.157, suggesting that the independent variables in the model could explain 15.7% of the total anxiety variance (*p* < 0.001).

## Discussion

The present cross-sectional study aimed to determine the relationship between spiritual health and COVID-19 anxiety among Iranian nurses. The findings revealed a reverse significant relationship between spiritual health and COVID-19 anxiety. On the other hand, nurses with better spiritual health experienced a lower level of COVID-19 anxiety.

These findings were similar to the findings of previous studies. For example, a previous study reported that spiritual health program improves sleep quality in Muslim stroke patients^28^. Another randomized trial study reported that group spiritual care program increased hope and reduced anxiety among leukemia patients^29^. A study reported that religious coping, and spiritual health were significantly related to reduced death anxiety in the elderly^30^. Another study reported a positive impact for spiritual care programs on reducing death anxiety among stroke patients^31^. Furthermore, a previous study reported a positive correlation between spiritual health and lower anxiety, and a good sleep quality among cancer patients^32^.

There is a large body of evidence that emphasize that religion and spirituality can promote mental health through positive religious coping, community and support, and positive beliefs^33^. Spiritual health alleviates the risk of mental disorders by integrating the forces and focus of the individual on solving psychological and social problems. When people regard themselves connected with a higher power, they can have more adaptation to their environmental conditions and will be less likely to suffer from mental disorders^34^.

A significant relationship has been reported between occupational stress and all the dimensions of spiritual health; however, the strongest relationship was observed between occupational stress and the existential dimension of spiritual health^11^. The results of another study that was conducted to assess the effect of religious intervention on the spiritual health of patients with anxiety disorder showed a significant inverse relationship between the level of spiritual health and the level of anxiety^35^. Furthermore, another study showed a significant relationship between spiritual health and internalizing coping strategies in patients, and patients with better spiritual health experienced a lower level of anxiety^36^.

The results of the current study indicated a high level of COVID-19 anxiety in Iranian nurses. Similarly, a previous study reported a high level of COVID-19 anxiety among Iranian nurses^37^. Another study reported that 37.8% of the Philipinian nurses had dysfunctional levels of anxiety related to COVID-19^38^. A previous study reported that the level of anxiety resulting from COVID-19 was higher in nurses compared to other occupation groups, and nurses were more exposed to mental illnesses^39^.

The results of the present study showed that anxiety was more prevalent among nurses with chronic diseases compared to those without such diseases. Soleimanzadeh et al. examined the relationship between spiritual health and death anxiety in older adults and indicated a high level of anxiety in older adults with underlying diseases and a significant relationship between spiritual health and anxiety^30^. These results were consistent with the results of the present study.

In the present study, anxiety was more prevalent among nurses with underlying diseases. Spiritual health is a basic concept concerning dealing with the problems and disease-related stress and integrates with other health dimensions. Low level of spiritual health has been linked to increased risk of psychological disorders such as sense of loneliness, anxiety, and loss of meaning in life. Strong spiritual health was reported to be able to effectively improve coping with disease among patients^40^.

## Strength and limitations

One of the strengths of the present study was that te study included nurses from the entire Iranian nursing population, which enhances the generalizability of the results. However, the limitation of the present study was the use of that self-report measures, which increased the risk of error and reduced the measurement precision. Also, personal differences can affect participants’ understanding of spiritual health and anxiety, but these factors could not be controlled by the researcher.

Spirituality health is a universal issue and is separated from religion. Although all of our participants in the study were Muslim, all religions have a strong spiritual component. Therefore, we believe that our findings can be generalized to all nurses around the world.

Another limitation of the present study was the inability of cross-sectional studies to establish causal relationships.

## Conclusion

The results showed a high level of COVID-19 anxiety in Iranian nurses. Moreover, the level of COVID-19 anxiety was lower in nurses with a higher spiritual health. Accordingly, it is recommended that measures be taken to maintain and promote nurses’ spiritual health. Strengthening spiritual health as a powerful force on physical, mental, and social health, can empower nurses to control COVID-19-related anxiety. In addition, by designing and presenting educational packages to enhance nurses’ spiritual health, steps can be taken to promote their spiritual health.

## Implications for management practice

Healthcare organizations and nursing administrations should develop strategies to protect nurses from the threat of COVID-19-related anxiety, which may improve the quality of care. A helpful strategy for this objective can be improving spiritual health. Future researches are recommended to examine the effect of interventions for improving spiritual health on COVID-19-related anxiety.

## Data Availability

The datasets used and/or analyzed during the current study are available from the corresponding author upon reasonable request.
