# Author Correction: The relationship between spiritual health and COVID-19 anxiety among nurses: a national online cross-sectional study

**DOI:** 10.1038/s41598-024-70483-7

**Published:** 2024-08-22

**Authors:** Arezoo Davarinia Motlagh Quchan, Fatemeh Mohammadzadeh, Zohreh Mohamadzadeh Tabrizi, Narjes Bahri

**Affiliations:** 1 Department of Health in Emergencies and Disasters, School of Public Health, Tehran University of Medical Sciences, Tehran, Iran; 2 Department of Epidemiology & Biostatistics, School of Health, Social Development & Health Promotion Research Center, Gonabad University of Medical Sciences, Gonabad, Iran; 3 Department of Nursing, School of Nursing and Midwifery, Mashhad University of Medical Sciences, Mashhad, Iran; 4 Department of Midwifery, Faculty of Medicine, Social Determinants of Health Research Center, Gonabad University of Medical Sciences, Gonabad, Iran

Correction to: *Scientific Reports* 10.1038/s41598-024-67523-7, published online 16 July 2024

In the original version of this Article Zohreh Mohamadzadeh Tabrizi was incorrectly affiliated with ‘Department of Health in Emergencies and Disasters, School of Public Health, Tehran University of Medical Sciences, Tehran, Iran’. The correct affiliation is listed below.

Department of Nursing, School of Nursing and Midwifery, Mashhad University of Medical Sciences, Mashhad, Iran.

The original Article has been corrected.

